# Flavonoids and Other Polyphenols: Bioactive Molecules from Traditional Medicine Recipes/Medicinal Plants and Their Potential for Phytopharmaceutical and Medical Application

**DOI:** 10.3390/molecules29235760

**Published:** 2024-12-05

**Authors:** Aekkhaluck Intharuksa, Sompop Kuljarusnont, Yohei Sasaki, Duangjai Tungmunnithum

**Affiliations:** 1 Department of Pharmaceutical Sciences, Faculty of Pharmacy, Chiang Mai University, Chiang Mai 50200, Thailand; aekkhaluck.int@cmu.ac.th; 2 Department of Obstetrics and Gynecology, Faculty of Medicine Siriraj Hospital, Mahidol University, Bangkok 10700, Thailand; sompop.kul@mahidol.edu; 3 Division of Pharmaceutical Sciences, Graduate School of Medical Plant Sciences, Kanazawa University, Kanazawa 920-1192, Japan; sasaki@p.kanazawa-u.ac.jp; 4 Department of Pharmaceutical Botany, Faculty of Pharmacy, Mahidol University, Bangkok 10400, Thailand; 5 Le Studium Institute for Advanced Studies, 1 Rue Dupanloup, 45000 Orléans, France

**Keywords:** flavonoids, polyphenols, traditional medicine, medicinal plants, medical application, pharmaceutical application

## Abstract

Currently, natural bioactive ingredients and/or raw materials are of significant interest to scientists around the world. Flavonoids and other polyphenols are a major group of phytochemicals that have been researched and noted as bioactive molecules. They offer several pharmacological and medical benefits. This current review aims to (1) illustrate their benefits for human health, such as antioxidant, anti-aging, anti-cancer, anti-inflammatory, anti-microbial, cardioprotective, neuroprotective, and UV-protective effects, and also (2) to perform a quality evaluation of traditional medicines for future application. Consequently, keywords were searched on Scopus, Google Scholar, and PubMed so as to search for related publications. Then, those publications were carefully checked in order to find current and non-redundant studies that matched the objective of this review. According to this review, researchers worldwide are very interested in discovering the potential of flavonoids and other polyphenols, used in traditional medicines and taken from medicinal plants, in relation to medical and pharmaceutical applications. Many studies focus on the health benefits of flavonoids and other polyphenols have been tested using in silico, in vitro, and in vivo models. However, few studies have been carried out using clinical trials that have trustworthy subject sizes and are in accordance with clinical practice guidelines. Additionally, interesting research directions and perspectives for future studies are highlighted in this work.

## 1. Introduction

**Figure 1:**
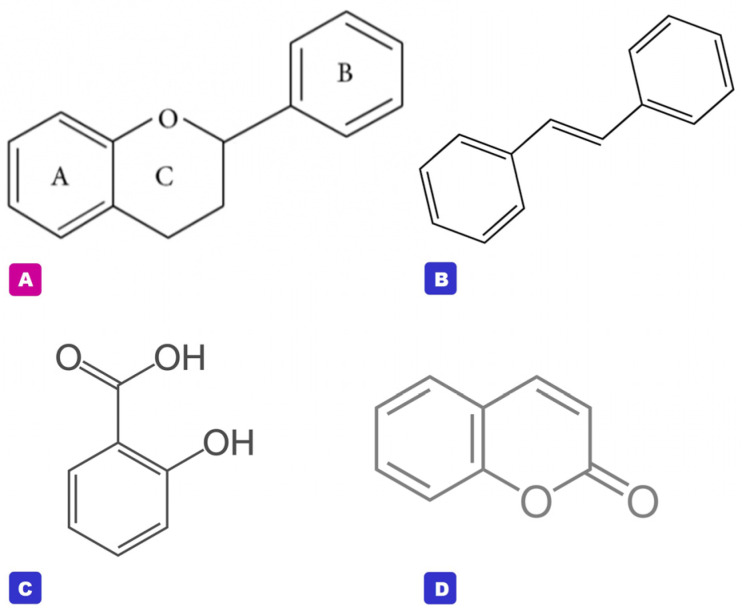
General structures of flavonoids (**A**) and other polyphenols (non-flavonoids, i.e., coumarins (**B**), phenolic acids (**C**), and stilbenes (**D**)).

In modern medical/phytopharmaceutical product development, the customers, researchers, and medical/pharmaceutical industrial sectors are interested in products developed from natural bioactive ingredients and/or raw materials taken from nature. These are used for the treatment and prevention of diseases, and to promote good health and well-being [1,2,3,4,5,6,7,8]. Flavonoids and other polyphenols are a large group of plant secondary metabolites or so-called phytochemicals that have been researched and noted as potential bioactive molecules with several pharmacological and medical benefits, such as antioxidant, anti-aging, anti-cancer, anti-inflammatory, anti-microbial, cardioprotective, neuroprotective, and UV-protective effects [4,5,6,7,8,9,10,11]. Flavonoids (Figure 1A) contain 2 aromatic rings (A and B rings) and linked by a C-3 chain to the oxygenated heterocyclic ring (C ring), whereas other polyphenols (Figure 1B–D) are compounds derived from shikimate and/or phenylpropanoid pathways, i.e., phenolic acids, coumarins, and stilbenes [12,13].

**Figure 2:**
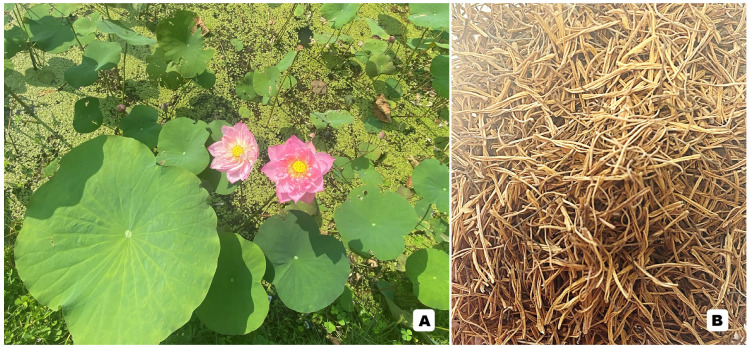
*Nelumbo nucifera* Gaertn., an example of a medicinal plant and traditional medicinal recipe: (**A**) *N. nucifera* medicinal plants in their aquatic natural habitat; (**B**) Thai traditional medicinal recipe made from the dried stamens of *N. nucifera*. All photos are taken by Assoc. Prof. Dr. Duangjai Tungmunnithum.

These phytochemical compounds have been reported extensively, not only in several species of medicinal plant but also in traditional medicinal recipes (Figure 2). This is especially true in Asian countries such as China, Japan, Thailand, and so on [4,8,14,15,16,17,18,19,20,21,22,23,24,25,26]. In the recent decade, more than 120,000 research studies have focused on flavonoids and other polyphenols according to the PubMed database (searched on 22 October 2024). It can undoubtedly be seen that both flavonoids and other polyphenols, taken from traditional medicines as well as medicinal plants, are of interest to researchers, who seek to discover their potential for medical and pharmaceutical applications [9,10,11,23,27,28,29,30,31,32,33,34,35,36,37]. This work intends to illustrate the potential of flavonoids and other polyphenols, taken from both traditional medicine recipes and medicinal plants, to provide antioxidant, anti-aging, anti-cancer, anti-inflammatory, anti-microbial, cardioprotective, neuroprotective, and UV-protective effects. Furthermore, the quality evaluation of traditional medicines, which are another source of flavonoids and other polyphenols, is also performed for the first time for use in future applications. Additionally, interesting research directions and perspectives for future studies are also highlighted in this recent review for use in future potential research work and applications.

## 2. Health Benefits of Flavonoids and Other Polyphenols Bioactive Molecules from Traditional Medicine Recipes and Medicinal Plants

### 2.1. Antioxidant Effect

It is commonly known that various cancer types, heart disease, diabetes, many other chronic diseases, and degenerative disorders can be caused by oxidative stress [19,38,39,40,41,42,43,44]. Unbalanced levels of reactive oxygen species (ROS) and reactive nitrogen species (RNS) are the by-products produced during adenosine triphosphate (ATP) production to produce energy for cells under oxygenic conditions [45,46,47,48]. This oxidative stress condition leads to various types of disease, such as diabetes, cancers, cardiovascular diseases, neurological diseases, chronic oxidative stress, inflammation-related diseases, etc. [45,46,47,48,49,50,51]. Flavonoids and other polyphenols have been researched worldwide in order to apply their potential to reduce excess ROS and RNS levels [9,10,11,27,28,29,36,37,52].

Li and his team [23] researched the effect of puerarin or 4′-7-dihydroxy-8-beta-d-glucosylisoflavone, which is a well-known flavonoid from the root of the Chinese medicinal plant known as *Pueraria lobate* (Wild) Ohwi, on Chinese participants with polycystic ovary syndrome (PCOS). This plant has become a synonym for *Pueraria montana* var. *lobata* (Wild.), as outlined in Maesen and S. M. Almeida and Sanjappa and Predeep, who searched the updated database known as “world flora online (WFO)”, https://www.worldfloraonline.org/, accessed on 22 October 2024. According to this randomized trial [23], a group of Chinese PCOS patients who received puerarin tablets of 150 mg/d orally for 3 months received antioxidant benefits from this flavonoid compared with the control group. This was established by measuring the antioxidant parameters, e.g., glutathione peroxidase, superoxide dismutase, catalase, etc. For example, a group of patients with PCOS who were treated with puerarin showed significantly different biomarkers in relation to antioxidative activities compared with the group of patients who did not take puerarin (*p* < 0.001). The antioxidant effect of puerarin taken from the *P. montana* medicinal plant was also confirmed in an in vivo animal model [53]. The study of Hou et al. [54] also showed that puerarin enhanced the antioxidant system through the regulation of adenosine monophosphate-activated protein kinase or the AMPK pathway. Moreover, the potential of this flavonoid antioxidant as a scavenging agent against reactive oxygen species was also reported by Zhu et al. [55]. Additionally, the effects of selected antioxidant agents, such as 3,4′,5-trihydroxystilbene (a resveratrol polyphenol), 3′,4′,5,7-tetrahydroxyflavone (a luteolin flavonoid) and 3,3′,4′,7-tetrahydroxyflavone (a fisetin flavonoid), on Gulf War Illness, i.e., a chronic multi-symptom disease of unknown etiology, were determined by the team of Hodgin [56] via a placebo-controlled, pseudo-randomized clinical trial study. The results showed that resveratrol is the most beneficial antioxidant agent among the 3 compounds tested and may help to reduce the symptoms of this illness [56].

The research study of Mendes et al. [57] focused on phlorizin and phloretin and discovered that both polyphenols displayed antioxidant potential by conducting an in silico molecular modeling assessment. In addition, the antioxidant effect of phlorizin was determined by using the in vivo *Caenorhabditis elegans* model [58]. The results suggested that phlorizin offers strong antioxidant ability via its oxidative stress response and autophagy and has potential to be used to develop a novel antioxidant nutraceutical for age-related diseases [58]. Interestingly, there are many research teams that are also interested in research on flavonoid-rich foods/beverages to treat or prevent the disease [30,31,59,60,61]. For example, Ismaeel et al. [61] determined the effect of flavanol-rich cocoa on the pathophysiology of muscle damage in peripheral artery disease (PAD), which is related to the increase in oxidant production and the decrease in the antioxidant defenses of PAD patients. This study pointed out that flavanol-rich cocoa beverages can activate nuclear factor erythroid 2-related factor 2 (Nrf2), help to increase the level of antioxidant protein, and protect skeletal muscle damage. This may result in improving the PAD patient’s walking performance [61]. Another study also reported on the antioxidant properties of epicatechin and quercetin phytochemical compounds. This team also showed that the combination of these 2 phytochemicals changed the cytoplasmic redox ambient and significantly improved biochemical parameters that are related to metabolic disorders [60]. 

### 2.2. Anti-Aging Effect

Aging is a kind of the time-related deterioration that can be indicated by various kinds of signaling, e.g., cellular senescence, deregulated nutrient sensing, DNA and mitochondrial dysfunction, telomere attrition, proteostasis loss, and so forth [62,63,64,65]. Aging brings a large number of undesirable effects to humanity, especially a decrease in quality of life and the beginning of several aging-associated diseases. The flavonoids and other polyphenols are interesting choices that many scientists worldwide have long researched regarding anti-aging properties, and medicinal plants as well as traditional medicinal recipes are the natural sources that scientists employ in several studies [66,67,68,69,70,71,72]. 

Chiu et al. focused their study on the clinical properties of roselle beverages made from *Hibiscus sabdariffa* L. or the so-called roselle plant [72]. This rich-polyphenol roselle beverage mainly consisted of 1.96 g of phenolics (gallic acid equivalent/100 mL) and 1.65 g of anthocyanins (cyanidin-3-glucoside equivalent/100 mL) and was employed in a clinical study (a randomized, cross-over, double-blind, placebo-controlled trial) involving 39 participants who were healthy adults for 6 months [72]. The results suggested that this rich polyphenol roselle beverage helped the treatment group’s participants to have lower blood pressure, controlled skin moisture and redness, and also improved antioxidation capacity [72]. Consequently, this team suggested it for use in beverage, phytopharmaceutical, and cosmetic applications [72]. Another randomized controlled trial was conducted using a fermented bilberry (*Vaccinium myrtillus*) extract, containing flavonoids and polyphenols [71], to illustrate their anti-aging and skin-brightening properties on 66 participants for 84 days. At day 84, those parameters were statistically significant in relation to the group of subjects who received fermented bilberry extract and those who received the placebo. The wrinkle depth decreased by 10.6%, firmness improved by 13.3%, elasticity improved by 12.4%, skin antioxidant capacity increased by 20.8%, and skin coloration increased by 20.8% [71].

The anti-aging potential of quercetin-3-*O*-rhamnoside and kaempferol-3-*O*-galactoside, which were the two most abundant flavonoids found in stamen extracts of *Nymphaea lotus* L. (Nymphaeaceae family), also known as an Asian water lily, was confirmed via both in silico molecular docking and in vitro enzymatic assays, i.e., elastase, collagenase, and tyrosinase [67]. The results showed that quercetin-3-*O*-rhamnoside plays an important role as an anti-collagenase with 60.24 ± 7.59% inhibition and as an anti-elastase with 50.28 ± 7.24% inhibition compared with the controls [67]. Kaempferol-3-*O*-galactoside displayed 59.84 ± 8.13% inhibition for collagenase and 55.56 ± 7.56% inhibition for elastase. This supported the molecular docking results, showing that quercetin-3-*O*-rhamnoside and kaempferol-3-*O*-galactoside showed superior binding affinity to the target enzymes compared to positive controls. Moreover, the computational predictions suggested that there was not severe toxicity for these 2 flavonoids [67]. The skin anti-aging properties of the 7 main flavonoids taken from *Nelumbo nucifera* Gaertn. were evaluated using molecular docking, and the most abundant kaempferol-3-*O*-robinobioside found in *N. nucifera* was evaluated using in vitro aging-related enzymes, e.g., tyrosinase, collagenase, and elastase [66]. Among the 7 flavonoids, kaempferol-3-*O*-robinobioside was the most promising candidate, exhibiting the highest docking scores for 3 enzymes. In addition, enzyme-based assays confirmed that kaempferol-3-*O*-robinobioside exhibited strong inhibitory effects against collagenase (58.24 ± 8.27% inhibition), elastase (26.29 ± 7.16% inhibition), and tyrosinase (69.84 ± 6.07% inhibition), respectively [66]. Additionally, Zhuang et al. [73] also examined the anti-aging effects of flavonoid-rich root extracts of *Nymphaea hybrid* in an in vivo animal model using *Caenorhabditis elegans.* The result showed that the extract significantly helped to extend *C. elegans*’s lifespan, which was probably associated with the insulin/IGF signaling pathway. This research team also discovered that the flavones present in the root extract of *N. hybrid* helped to increase the survival rate of *C. elegans* in both normal and stress conditions, suggesting that this flavonoid-rich extract could be used for the development of functional food [73]. Another anti-aging effect of the *Nymphaea* medicinal plant was achieved by Park at al. on *N. tetragona* Georgi, the distribution of which is ranging from Europe to Korea, including the Himalayas [74]. The results demonstrated that the use of rhizome extracts from this medicinal plant species decreased mitochondrial dysfunction, reactive oxygen species production, Bcl-2-associated X protein levels, and the release of cytochrome c from mitochondria [74]. This study also showed that the rhizomatous extract protected skin aging by mediating anti-apoptosis via reactive oxygen species scavenging in human epidermal keratinocytes [74]. 

The 4′,5,7-trihydroxyflavone or apigenin was determined to have anti-aging effects in vitro using normal human dermal fibroblasts cells and in a clinical study on the skin anti-aging effects of an apigenin-containing cream [70]. This study found that apigenin-containing cream helped to reduce fine wrinkles and increase dermal elasticity, density, skin evenness, and moisture content in skin, and apigenin was suggested by this team as an anti-aging ingredient for use in cosmetics [70]. Another research group also determined the skin anti-aging effects of oleacein via a randomized, single-blind clinical trial on 70 participants, and recommended the use of oleacein as a potential ingredient for anti-aging skin care [69]. The in vivo aging mouse model induced by d-galactose was used to investigate the effect of lotus medicinal plant seedpod proanthocyanidins (LSPCs) on brain aging and cognitive impairment [68]. The results showed that LSPCs significantly improved learning and memory and reduced the nitric oxide and malondialdehyde levels in this aging mouse model. This study also found that LSPCs could prevent neuron damage and decreased P53 protein expression in animal models, which was helpful for Alzheimer’s disease treatment [68].

### 2.3. Anti-Cancer Effect

According to the GLOBOCAN online database published in this year [75], the cancer case number/year will increase sharply and reach 35 million in the year 2050. Investment in cancer prevention is strongly suggested in order to save many millions of lives worldwide from this serious disease [75,76]. Furthermore, the current treatments mainly focus on chemotherapy, surgery, and radiotherapy, which are applied with some additional approaches such as hormonal therapy, immunotherapy, and targeted therapy [75,76]. Unfortunately, these cancer therapeutics are linked to unintended side effects, and probably cause people to develop resistance to anti-cancer medicines. Subsequently, we are searching for alternative treatment methods, with no/fewer adverse side effects, to prevent or fight against cancers. In the current decade, the bioactive compounds from several medicinal plant have been researched for treatment and prevention, and to decrease undesirable side effects [77,78,79,80,81,82,83,84]. 

Additionally, phytopharmaceutical products/dietary supplements are also helpful for the therapeutic treatment of many cancer types [77,80,84]. Likewise, rutin, a flavonoid found in the leaf extract of *Murraya koenigii* L. [the correct species name of this medicinal plant is *Bergera koenigii* L. (https://powo.science.kew.org/taxon/urn:lsid:ipni.org:names:771522-1, accessed on 22 October 2024)], is an anti-cancer agent that fights against breast cancer, as tested on the MDA-MB-231 cancer cell line [77]. Ahmed et al. [80] also reported on the anti-cancer potential of flavonoids taken from leaves extract of *Cassia angustifolia* Vahl. [the correct species name of this medicinal plant is *Senna alexandrina* var. *alexandrina* (https://powo.science.kew.org/taxon/urn:lsid:ipni.org:names:77208460-1, accessed on 22 October 2024)]. Lee et al. examined the anti-pancreatic cancer effects of the combination of naringenin and hesperetin from *Citrus unshiu* peel via in vitro and in vivo models [84]. The results indicated that administration of hesperetin and naringenin inhibited the migration of human pancreatic cancer via the downregulation of focal adhesion kinase and the p38 signaling pathway [84].

The anti-cancer effect of epigallocatechin-3-gallate, which is a well-known flavonoid from extracted *Camellia sinensis* (L.) Kuntze or green tea, was also studied. Xia et al. investigated the safety and efficacy of epigallocatechin-3-gallate for the treatment of acute severe dermatitis in thoracic cancer patients with radiotherapy in the clinical trial Phase I [83]. The epigallocatechin-3-gallate solution was sprayed onto the radiation field area when grade III radiation-induced dermatitis occurred for the first time [83]. The results from this study showed that no radiation therapy delay and no adverse events were observed in all 19 patients, and it was recommended that the highest dose (2574 μmol/L) from the Phase I trial be continued in the Phase II trial [83]. This team also suggested epigallocatechin-3-gallate as a potential choice for acute severe radiation-induced dermatitis [83].

Traditional medicine/traditional medicinal recipes have also been studied in this recent decade to discover potential choices of drugs for cancer patients. Shao et al. [82] examined the effect of traditional Chinese medicine, *Portulaca oleracea* L., on digestive inflammatory cancer transformation. This study indicated that apigenin, lupeol, luteolin, myricetin, genistein, and quercetin were the potential flavonoids that could act against colitis and colitis-associated cancers [82]. However, the mechanism of action of these flavonoids in the colitis-associated cancers was not determined in this study, and so the authors suggested that future research be carried out to clinically study the mode of action and pharmacokinetic data. Chen and his team [81] aimed to examine the antitumor efficacy of the Yishen Qutong granule (YSQTG) in primary lung cancer treatment, and identified the key active pharmaceutical ingredients (APIs). They also explored its mechanism of action. The Yishen Qutong granule was reported to have anti-lung cancer potential due to its inhibition of lung cancer cell proliferation. It also induced apoptosis in the vitro model and inhibited tumor growth in the lungs in an in vivo animal model by the downregulation of oxidative stress-related HMOX1 protein expression [81].

### 2.4. Anti-Inflammatory Effect 

Inflammation is generally known as a part of the body’s defense mechanism, functioning as part of the body’s immune system to deal with foreign stimuli, fight off infection, or heal damaged cells/tissues [85,86,87,88]. Inflammation is mainly classified into acute or chronic types depending on the period of inflammation, the type of stimuli, and also the ability of the body to repair and overcome that inflammatory damage [85,89,90]. Chronic inflammation, which mostly occurs in long-term periods ranging from many months to years, induces more undesirable results in the body and health conditions such as autoimmune disorders, auto-inflammatory disorders, mitochondrial dysfunction, and oxidative stress conditions, e.g., the excess production of free radicals, oxidized lipoproteins, advanced glycation end products (AGEs), and so forth [87,91,92,93]. This leads to unwanted chronic inflammatory diseases: cancer, diabetes, obesity, heart disorders, stroke, chronic respiratory diseases, and many others [86,89,90,94]. Groups of natural bioactive molecules like flavonoids and other polyphenols are among the most interesting choices for the treatment and/or prevention of inflammation and its related illnesses [95,96,97,98,99,100,101,102,103,104,105,106,107,108], and research is being conducted to discover their pharmacological activities/medical benefits.

The anti-inflammatory effects of polyphenols from *Forsythia suspensa* (Thunb.) Vahl and their mechanisms of action on inflammatory bowel disease were investigated [108]. The results revealed that the polyphenols from *F. suspensa* inhibited M1 polarization apoptosis and pyroptosis, and also regulated intestinal homeostasis in in vivo mouse models [108]. This study also reported that the polyphenols helped to improve gut microbiota and enhance intestinal metabolites’ short-chain fatty acid levels [108]. Interestingly, a recent study also supported the potential of flavonoids in relation to inflammatory bowel disease [103]. The study examined the protective effects of apigenin and epigallocatechin-3-gallate combinations; the in vitro results from Caco-2 cell monolayers indicated that the combination of these 2 flavonoids displayed protective effects on the intestinal epithelium’s barrier function [103]. Conversely, the in vivo results showed that the combination of apigenin and epigallocatechin-3-gallate pointedly decreased inflammatory levels in both chronic and acute hapten-mediated experimental colitis models in a dose-dependent and time-dependent manner [103].

Likewise, the effect of inflammatory dermatosis and the mechanism of polyphenol-rich extracts from *Centella asiatica* (L.) Urb., a medicinal plant of the Umbelliferae family, have been examined by Lin et al. using both in vitro HaCaT cells induced by 20 μg·mL^−1^ lipopolysaccharide and an in vivo mouse model of imiquimod-induced psoriasis-like skin inflammation [107]. The results showed that the *C. asiatica* extract downregulated mRNA levels of inflammatory factors such as IFN-γ, IL-6, CCL20, and TNF-α as well as improved the gene expression of barrier-protective factors [107]. Using 25 μg·mL^−1^ of extract also inhibited NF-κB and JAK/STAT3 pathway activation [107]. The in vivo results indicated that 40 mg·mL^−1^ of extract helped to inhibit inflammatory factor secretion in serum as well as skin lesions, and to reduce blood scabbing and skin scaling [107]. So, this team suggested *C. asiatica* extract as an ingredient for skin care and functional food products [107]. Another research group reported on the anti-inflammatory properties of polyphenol from black chokeberry, i.e., *Aronia melanocarpa*, a medicinal plant from the Rosaceae family in temperate biomes, on obesity-induced colonic inflammation in a high-fat-diet-fed rat model as well as the lipopolysaccharide-stimulated inflammatory response using RAW264.7 cells [106]. The findings indicated that polyphenols from black chokeberry decreased nitric oxide production and TNF-α, IL-6, IL-1β, and MCP-1 pro-inflammatory cytokine production in LPS-induced RAW264.7 cells. This polyphenol supplementation also helped to improve glucose tolerance and attenuate systemic inflammation, improve intestinal barrier dysfunction, and also suppress mRNA expression of pro-inflammatory cytokines in a high-fat-diet-fed rat model [106].

Moreover, the anti-inflammatory activity of the flavonoids from *Smilax china* L. (Smilacaceae family), a traditional medicinal plant species used in China, was examined via an in vivo high-fat/high-sucrose-induced obese mouse model for 3 months [99]. The results showed that flavonoids from *S. china* effectively inhibited high-fat/high-sucrose-induced obesity in mice by the suppression of lipopolysaccharide-producing bacteria as well as pro-inflammatory bacteria [99]. This team suggested flavonoids from *S. china* as a hopeful prophylactic for diet-induced inflammatory illnesses via the gut–liver axis [99]. Another research study emphasized the anti-inflammatory potential and the mechanism of kaempferol in an obesity mouse model, and suggested this flavonoid as a dietary supplement for the treatment of obesity [98]. The results illustrated that kaempferol supplementation could improve the intestinal barrier, attenuate gut inflammation, and counteract dysbiosis related to obesity in high-fat-diet mice [98]. Additionally, another study reported on the therapeutic effect of kaempferol on skin inflammation by using in vivo MC903-induced atopic dermatitis-like skin inflammation mice [96]. This team demonstrated that kaempferol suppressed the expression of MC903-induced dermatitis, thymic stromal lymphopoietin, transepidermal water loss, and heme-oxygenase-1, and also enhanced barrier dysfunction in the treated mouse model [96]. So, they proposed kaempferol as a new treatment choice for atopic dermatitis [96]. Remarkably, traditional medicine is also of interest to researchers seeking to discover its potential in anti-inflammation. An obvious example is Wumei Wan, a traditional Chinese medicine that has long been used for digestive disorders and which is suggested for anti-colitis treatment [98]. Kaempferol, taxifolin, luteolin, and quercetin were also determined to be bioactive flavonoids present in this traditional Chinese medicine [98]. The results from network pharmacology and in vivo analysis indicated that Wumei Wan helped to improve dextran sulfate sodium salt-induced colitis and attenuate inflammatory cytokine as well as chemokine expression [95]. 

Additionally, a study on the anti-inflammatory effects of galangin also reported on its ability to effectively prevent the nephrotoxicity-related side effects of gentamicin, an antibiotic drug [109]. This work was conducted on an in vivo rat model in which the treatment group received galangin for 14 days and received gentamicin from day 8 to day 14 [109]. The results showed that galangin prevented tissue injury, reduced malondialdehyde and nitric oxide levels, and reduced inflammatory mediator levels in the kidney of the treatment group [109]. The therapeutic effects of sakuranetin, a cherry flavonoid, on chondrocyte inflammation and chondrogenesis process in osteoarthritis were investigated using an in vivo osteoarthritis rat model [102]. The study indicated that the cherry flavonoid sakuranetin helped to improve chondrocyte inflammation, and also promoted chondrogenesis by the PI3K/AKT/NF-κB pathway inhibition [102]. Furthermore, formononetin, which is an isoflavone phytoestrogen, has recently been investigated and suggested as an alternative asthma treatment [100]. This research group conducted an in vivo animal test to determine the potential effect of formononetin on airway inflammation as well as epithelial barrier repair by using house dust mite-induced asthmatic mice [100]. The result proved that the flavonoid formononetin inhibited both Toll-like receptor 4 and estrogen receptor/Nod-like receptor family pyrin domain-containing protein 3/Caspase-1 signaling, resulting in a decrease in airway inflammation in mice models [100].

### 2.5. Anti-Microbial Effect

Several scientists are interested in plants because they are a rich source of phytochemicals with a variety of biological activities. Flavonoids and polyphenolics are known as some of the phytochemical groups which provide inhibitory effects against microorganisms. Pathogenic microorganisms, namely viruses, fungi, yeasts, and especially bacteria, are hazardous for human health. It is not surprising that flavonoids and other polyphenols have recently been revealed to possess antibacterial activities in several scientific studies (Table 1). As we know, bacteria can transfer to humans through foodborne transmission, airborne transmission, droplet transmission, and physical contact. In cases of foodborne transmission, spoilage and exposure to pathogenic bacteria in the production line are potential vectors of disease in the food industry [110]. There are many bacteria caused by foodborne illnesses, namely, *Campylobacter jejuni*, *Escherichia coli* 0157:H7, *Listeria monocytogenes*, *Salmonella* spp., and *Staphylococcus aureus* [110,111]. 

Foodborne disease can bring about high morbidity and mortality rates [112]. Synthetic, semi-synthetic, and natural antibacterial substances were chosen to inhibit the growth of bacteria. However, plant-based preservatives have been discussed and considered as potential alternatives to chemical preservatives that are detrimental to human health and increase the risk of cancer, methemoglobinemia, hypersensitivity, and allergy [113]. As described in Table 1, natural flavonoids and polyphenols exhibit inhibitory activity against foodborne bacteria. For example, phenolics which were extracted from wine residues showed antibacterial activity against *Escherichia coli* 0157:H7 [114]. Barreca et al. revealed that phloretin, a dihydrochalcone found in apple and kumquat, could inhibit the growth of *Listeria monocytogenes* ATCC 13932 in the microbroth dilution method [115]. In 2019, Ma et al.’s study focused on the antibacterial properties of anthocyanins and catechins against *E. coli* and *Salmonella*, common foodborne pathogens. They found that anthocyanins and catechins could significantly decrease the abundance of pathogenic bacteria [112]. Furthermore, (−)-Epigallocatechin-3-gallate (EGCG), an important active compound in green tea, exhibited antibacterial activity against a wide-spectrum of both Gram-positive and Gram-negative bacteria responsible for food spoilage. 

In other pathogenic transmission cases, bacteria are also potential dangers to human health. In in vitro studies, there are many flavonoids and phenolics that are effective, displaying broad-spectrum antibacterial activity (Table 1). For example, quercetin showed antibacterial effects against *Staphylococcus aureus* ATCC 25923 [116], *S. aureus* ATCC 6538P [116], *S. aureus* MTCC-740 [117], *S. aureus* NCTC 12900 [114], *Escherichia coli* ATCC 25922 [118], *E. coli* MTCC-119 [117], *E. coli* 0157:H7 [114], *Klebsiella pneumonia* subsp. *pneumoniae* MTCC-109 [117], and *Salmonella enterica* ssp. *enterica* ATCC BAA-2162 [116]. Epicatechin can fight against *Staphylococcus aureus* ATCC 25923 [116], *S. aureus* ATCC 6538P [116], *S. aureus* ATCC 29213 [119], *Escherichia coli* ATCC 8739 [116], and *Salmonella enterica* ssp. *enterica* ATCC BAA-2162 [116]. Additionally, multidrug-resistant bacteria are threats to public health around the world. Therefore, the discovery of effective antibiotics against these bacteria is urgently necessary. Wu et al. studied the antibacterial effect of flavonoids extracted from licorice against methicillin-resistant *Staphylococcus aureus* (MRSA). They found that glabrol, licochalcone A, licochalcone C, and licochacone C successfully combated MRSA [120]. Quercetin-3-*O*-rutinoside and kaempferol-3-*O*-rutinoside exhibited the potential to inhibit the growth of *β*-lactamase-producing *Klebsiella pneumoniae* [121]. Moreover, the combination of norfloxacin and genistein extracted from *Sophora moorcroftiana* showed significantly synergistic effects against the drug-resistant *Staphylococcus aureus* strain SA1199B [122]. As antibacterial agents, flavonoids, and polyphenolics probably counter bacteria through many mechanisms of action, such as the destruction of the bacterial cell wall and cell membrane, the inhibition of biofilm formation, the inhibition of bacterial enzymes and substrate deprivation, the deprivation of metal iron, and protein regulation [110,113].

**Table 1:** Antimicrobial potential of flavonoids and other polyphenols from various sources.

Microorganisms	Flavonoids and Other Polyphenols	Sources	Methods	References
**Gram-positive**				
*Bacillus subtilis* ATCC 6633	Camaroside	*Calotropis procera* Ait.	Agar well diffusion	[123]
	Betmidin	*Lannea alata* (Engl.) Engl.	Disk diffusion	[124]
	Dihydrolanneaflavonol	*Lannea alata* (Engl.) Engl.	Disk diffusion	
	Narcissin	*Calotropis procera* Ait.	Agar well diffusion	[123]
	Nicotiflorin	*Calotropis procera* Ait.	Agar well diffusion	
	Lanneaflavonol	*Lannea alata* (Engl.) Engl.	Disk diffusion	[124]
	Myricitrin	*Lannea alata* (Engl.) Engl.	Disk diffusion	
	Rutin	*Calotropis procera* Ait.	Agar well diffusion	[123]
*Listeria monocytogenes* ATCC 13932	Phloretin	*Malus domestica* (Suckow) Borkh.	Microbroth dilution	[115]
*Listeria monocytogenes* F2365	Oleuropein	*Olea europaea* L.	Two-fold dilution	[125]
*Micrococcus lueus* ATCC 4698	Camaroside	*Calotropis procera* Ait.	Agar well diffusion	[123]
	Narcissin	*Calotropis procera* Ait.	Agar well diffusion	
	Nicotiflorin	*Calotropis procera* Ait.	Agar well diffusion	
	Rutin	*Calotropis procera* Ait.	Agar well diffusion	
*Staphylococcus aureus* CECT 976	Umbelliferone	Pure	Microbroth dilution	[126]
*Staphylococcus aureus* PCM 2054	4′-methoxychalcones	*Humulus lupulus* L.	Agar disk diffusion	[127]
	Naringenin chalcone	*Humulus lupulus* L.	Agar disk diffusion	
	Naringenin	*Humulus lupulus* L.	Agar disk diffusion	
	Xanthohumol	*Humulus lupulus* L.	Agar disk diffusion	
*Staphylococcus aureus* ATCC 25923	Camaroside	*Calotropis procera* Ait.	Agar well diffusion	[123]
	Chrysoeriol-7-*O*-*β*-d-apiofuranosyl-(1→2)-*β*-d-xylopyranoside	*Graptophyllum grandulosum* Turrill	Microbroth dilution	[128]
	Chrysoeriol-7-*O*-α-l-rhamnopyranosyl-(1→6)-*β*-d-glucopyranoside	*Graptophyllum grandulosum* Turrill	Microbroth dilution	
	Epicatechin	*Aronia melanocarpa* Michx.	Agar diffusion	[116]
	Isorhamnetin-3-*O*-α-l-rhamnopyranosyl-(1→6)-*β*-d-glucopyranoside	*Graptophyllum grandulosum* Turrill	Microbroth dilution	[128]
	Narcissin	*Calotropis procera* Ait.	Agar well diffusion	[123]
	Nicotiflorin	*Calotropis procera* Ait.	Agar well diffusion	
	Graveobioside	*Graptophyllum grandulosum* Turrill	Microbroth dilution	[128]
	Quercetin	*Aronia melanocarpa* Michx.	Agar diffusion	[116]
	Rutin	*Calotropis procera* Ait.	Agar well diffusion	[123]
*Staphylococcus aureus* ATCC 29213	Betmidin	*Lannea alata* (Engl.) Engl.	Disk diffusion	[124]
	Dihydrolanneaflavonol	*Lannea alata* (Engl.) Engl.	Disk diffusion	
	Lanneaflavonol	*Lannea alata* (Engl.) Engl.	Disk diffusion	
	Myricitrin	*Lannea alata* (Engl.) Engl.	Disk diffusion	
*Staphylococcus aureus* ATCC 43300	Betmidin	*Lannea alata* (Engl.) Engl.	Disk diffusion	
	Dihydrolanneaflavonol	*Lannea alata* (Engl.) Engl.	Disk diffusion	
	Lanneaflavonol	*Lannea alata* (Engl.) Engl.	Disk diffusion	
*Staphylococcus aureus* ATCC 6538	(3S)-Licoricidin	*Glycyrrhiza aspera* Pall.	Microbroth dilution	[129]
	Glycycoumarin	*Glycyrrhiza aspera* Pall.	Microbroth dilution	
	Licorisoflavan A	*Glycyrrhiza aspera* Pall.	Microbroth dilution	
	Phloretin	*Malus domestica* (Suckow) Borkh.	Microbroth dilution	[115]
	Phloretin 3′,5′-di-*C*-glucoside	*Malus domestica* (Suckow) Borkh.	Microbroth dilution	
	Phloridzin	*Malus domestica* (Suckow) Borkh.	Microbroth dilution	
	Topazolin	*Glycyrrhiza aspera* Pall.	Microbroth dilution	[129]
*Staphylococcus aureus* ATCC 6538P	Epicatechin	*Aronia melanocarpa* Michx.	Agar diffusion	[116]
	Quercetin	*Aronia melanocarpa* Michx.	Agar diffusion	
*Staphylococcus aureus* ATCC 29213	Epicatechin	*Ficus sansibarica* Warb. subsp. *sansibarica*	Agar well diffusion	[119]
*Staphylococcus aureus* MRSA	4-Hydroxylonchocarpin	*Dorstenia barteri* Bureau	Microbroth dilution	[130]
	6-Phenylapigenin	*Dorstenia dinklagei* Engl.	Microbroth dilution	
	6,8-Diprenyleriodictyol	*Dorstenia manii* Hook.f.	Microbroth dilution	
	Isobavachalcone	*Dorstenia barteri* Bureau	Microbroth dilution	
*Staphylococcus aureus* MTCC-740	Caffeic acid	*Syzygium cumini* (L.) Skeels.	Agar well diffusion	[117]
	Delphinidin chloride	*Syzygium cumini* (L.) Skeels.	Agar well diffusion	
	Gallic acid	*Syzygium cumini* (L.) Skeels.	Agar well diffusion	
	Quercetin	*Syzygium cumini* (L.) Skeels.	Agar well diffusion	
	Sinapic acid	*Syzygium cumini* (L.) Skeels.	Agar well diffusion	
*Staphylococcus aureus* NCTC 12900	Caffeic acid	*Vitis vinifera* L.	Microbroth dilution	[114]
	Catechin	*Vitis vinifera* L.	Microbroth dilution	
	Chlorogenic acid	*Vitis vinifera* L.	Microbroth dilution	
	Coumarin	*Vitis vinifera* L.	Microbroth dilution	
	Cyanidin chloride	*Vitis vinifera* L.	Microbroth dilution	
	Cyanin chloride	*Vitis vinifera* L.	Microbroth dilution	
	Delphinidin chloride	*Vitis vinifera* L.	Microbroth dilution	
	Gallic acid	*Vitis vinifera* L.	Microbroth dilution	
	Ideain chloride	*Vitis vinifera* L.	Microbroth dilution	
	Keracyanin chloride	*Vitis vinifera* L.	Microbroth dilution	
	Kuromanin chloride	*Vitis vinifera* L.	Microbroth dilution	
	Malvidin chloride	*Vitis vinifera* L.	Microbroth dilution	
	Pelargonidin chloride	*Vitis vinifera* L.	Microbroth dilution	
	Quercetin	*Vitis vinifera* L.	Microbroth dilution	
	Resveratrol	*Vitis vinifera* L.	Microbroth dilution	
	Syringic acid	*Vitis vinifera* L.	Microbroth dilution	
	Tannic acid	*Vitis vinifera* L.	Microbroth dilution	
*Staphylococcus epidermidis* ATCC 12228	Camaroside	*Calotropis procera* Ait.	Agar well diffusion	[123]
	Narcissin	*Calotropis procera* Ait.	Agar well diffusion	
	Nicotiflorin	*Calotropis procera* Ait.	Agar well diffusion	
	Rutin	*Calotropis procera* Ait.	Agar well diffusion	
*Staphylococcus epidermidis* ATCC 14990	Dihydrolanneaflavonol	*Lannea alata* (Engl.) Engl.	Disk diffusion	[124]
	Lanneaflavonol	*Lannea alata* (Engl.) Engl.	Disk diffusion	
*Staphylococcus saprophyticus* ATCC 35552	Betmidin	*Lannea alata* (Engl.) Engl.	Disk diffusion	
	Dihydrolanneaflavonol	*Lannea alata* (Engl.) Engl.	Disk diffusion	
	Lanneaflavonol	*Lannea alata* (Engl.) Engl.	Disk diffusion	
	Myricitrin	*Lannea alata* (Engl.) Engl.	Disk diffusion	
*Staphylococcus scuiri* ATCC 29062	Betmidin	*Lannea alata* (Engl.) Engl.	Disk diffusion	
	Dihydrolanneaflavonol	*Lannea alata* (Engl.) Engl.	Disk diffusion	
*Staphylococcus xylosus* ATCC 35033	Betmidin	*Lannea alata* (Engl.) Engl.	Disk diffusion	
	Dihydrolanneaflavonol	*Lannea alata* (Engl.) Engl.	Disk diffusion	
	Lanneaflavonol	*Lannea alata* (Engl.) Engl.	Disk diffusion	
	Myricitrin	*Lannea alata* (Engl.) Engl.	Disk diffusion	
*Staphylococcus. aureus* ATCC 25923	Chrysoeriol-7-*O*-*β*-d-xyloside	*Graptophyllum grandulosum* Turrill	Microbroth dilution	[128]
*Staphylococcus. scuiri* ATCC 29062	Lanneaflavonol	*Lannea alata* (Engl.) Engl.	Disk diffusion	[124]
*Streptococcus agalactiae* ATCC 13813	Myricitrin	*Lannea alata* (Engl.) Engl.	Disk diffusion	
*Streptococcus pyrogenes* ATCC 19615	Betmidin	*Lannea alata* (Engl.) Engl.	Disk diffusion	
	Dihydrolanneaflavonol	*Lannea alata* (Engl.) Engl.	Disk diffusion	
	Lanneaflavonol	*Lannea alata* (Engl.) Engl.	Disk diffusion	
	Myricitrin	*Lannea alata* (Engl.) Engl.	Disk diffusion	
**Gram-negative**				
*Escherichia coli* ATCC 25922	Kaempferol	Pure	Microbroth dilution	[118]
	Quercetin	Pure	Microbroth dilution	
	Chrysin	Pure	Microbroth dilution	
	Luteolin	Pure	Microbroth dilution	
	Baicalein	Pure	Microbroth dilution	
	Tangeritin	Pure	Microbroth dilution	
	Daidzein	Pure	Microbroth dilution	
	Genistin	Pure	Microbroth dilution	
	Puerarin	Pure	Microbroth dilution	
	Rutin	*Calotropis procera* Ait.	Agar well diffusion	[123]
	Nicotiflorin	*Calotropis procera* Ait.	Agar well diffusion	
	Camaroside	*Calotropis procera* Ait.	Agar well diffusion	
*Escherichia coli* ATCC 8739	(3S)-Licoricidin	*Glycyrrhiza aspera* Pall.	Microbroth dilution	[129]
	Licorisoflavan A	*Glycyrrhiza aspera* Pall.	Microbroth dilution	
	Topazolin	*Glycyrrhiza aspera* Pall.	Microbroth dilution	
	Glycycoumarin	*Glycyrrhiza aspera* Pall.	Microbroth dilution	
	Epicatechin	*Aronia melanocarpa* Michx.	Agar diffusion	[116]
*Escherichia coli* CECT 434	7-Hydroxycoumarin	Pure	Microbroth dilution	[126]
*Escherichia coli* MTCC-119	Gallic acid	*Syzygium cumini* (L.) Skeels.	Agar well diffusion	[117]
	Caffeic acid	*Syzygium cumini* (L.) Skeels.	Agar well diffusion	
	Sinapic acid	*Syzygium cumini* (L.) Skeels.	Agar well diffusion	
	Quercetin	*Syzygium cumini* (L.) Skeels.	Agar well diffusion	
	Delphinidin chloride	*Syzygium cumini* (L.) Skeels.	Agar well diffusion	
*Escherichia coli* 0157:H7	Oleuropein	*Olea europaea* L.	Two-fold dilution	[125]
	Gallic acid	*Vitis vinifera* L.	Microbroth dilution	[114]
	Syringic acid	*Vitis vinifera* L.	Microbroth dilution	
	Caffeic acid	*Vitis vinifera* L.	Microbroth dilution	
	Coumaric acid	*Vitis vinifera* L.	Microbroth dilution	
	Tannic acid	*Vitis vinifera* L.	Microbroth dilution	
	Chlorogenic acid	*Vitis vinifera* L.	Microbroth dilution	
	Resveratrol	*Vitis vinifera* L.	Microbroth dilution	
	Catechin	*Vitis vinifera* L.	Microbroth dilution	
	Keracyanin chloride	*Vitis vinifera* L.	Microbroth dilution	
	Kuromanin chloride	*Vitis vinifera* L.	Microbroth dilution	
	Delphinidin chloride	*Vitis vinifera* L.	Microbroth dilution	
	Cyanin chloride	*Vitis vinifera* L.	Microbroth dilution	
	Cyanidin chloride	*Vitis vinifera* L.	Microbroth dilution	
	Ideain chloride	*Vitis vinifera* L.	Microbroth dilution	
	Pelargonidin chloride	*Vitis vinifera* L.	Microbroth dilution	
	Malvidin chloride	*Vitis vinifera* L.	Microbroth dilution	
	Quercetin	*Vitis vinifera* L.	Microbroth dilution	
*Escherichia. coli* ATCC 25922	Ononin	Pure	Microbroth dilution	[118]
	Narcissin	*Calotropis procera* Ait.	Agar well diffusion	[123]
*Klebsiella pneumonia* subsp. *pneumoniae* MTCC-109	Gallic acid	*Syzygium cumini* (L.) Skeels.	Agar well diffusion	[117]
	Caffeic acid	*Syzygium cumini* (L.) Skeels.	Agar well diffusion	
	Sinapic acid	*Syzygium cumini* (L.) Skeels.	Agar well diffusion	
	Quercetin	*Syzygium cumini* (L.) Skeels.	Agar well diffusion	
	Delphinidin chloride	*Syzygium cumini* (L.) Skeels.	Agar well diffusion	
*Klebsiella pneumoniae* ATCC 13883	Rutin	*Calotropis procera* Ait.	Agar well diffusion	[123]
	Nicotiflorin	*Calotropis procera* Ait.	Agar well diffusion	
	Narcissin	*Calotropis procera* Ait.	Agar well diffusion	
	Camaroside	*Calotropis procera* Ait.	Agar well diffusion	
*Proteus vulgaris* NRRL B-123	Iconisaflavan	*Glycyrrhiza aspera* Pall.	Microbroth dilution	[129]
	Iconisoflaven	*Glycyrrhiza aspera* Pall.	Microbroth dilution	
	(3S)-Licoricidin	*Glycyrrhiza aspera* Pall.	Microbroth dilution	
	Licorisoflavan A	*Glycyrrhiza aspera* Pall.	Microbroth dilution	
	Topazolin	*Glycyrrhiza aspera* Pall.	Microbroth dilution	
	Glycycoumarin	*Glycyrrhiza aspera* Pall.	Microbroth dilution	
*Pseudomonas aeruginosa* ATCC 10145	Iconisaflavan	*Glycyrrhiza aspera* Pall.	Microbroth dilution	
	Iconisoflaven	*Glycyrrhiza aspera* Pall.	Microbroth dilution	
	(3S)-Licoricidin	*Glycyrrhiza aspera* Pall.	Microbroth dilution	
	Licorisoflavan A	*Glycyrrhiza aspera* Pall.	Microbroth dilution	
	Topazolin	*Glycyrrhiza aspera* Pall.	Microbroth dilution	
	Glycycoumarin	*Glycyrrhiza aspera* Pall.	Microbroth dilution	
*Pseudomonas aeruginosa* ATCC 27853	Lanneaflavonol	*Lannea alata* (Engl.) Engl.	Disk diffusion	[124]
	Rutin	*Calotropis procera* Ait.	Agar well diffusion	[123]
	Nicotiflorin	*Calotropis procera* Ait.	Agar well diffusion	
	Narcissin	*Calotropis procera* Ait.	Agar well diffusion	
	Camaroside	*Calotropis procera* Ait.	Agar well diffusion	
*Pseudomonas aeruginosa* ATCC 35032	Lanneaflavonol	*Lannea alata* (Engl.) Engl.	Disk diffusion	[124]
*Salmonella enterica* ssp. *enterica* ATCC BAA-2162	Epicatechin	*Aronia melanocarpa* Michx.	Agar diffusion	[116]
	Quercetin	*Aronia melanocarpa* Michx.	Agar diffusion	
*Salmonella enteritidis* ATCC 13076	Rutin	*Calotropis procera* Ait.	Agar well diffusion	[123]
	Nicotiflorin	*Calotropis procera* Ait.	Agar well diffusion	
	Narcissin	*Calotropis procera* Ait.	Agar well diffusion	
	Camaroside	*Calotropis procera* Ait.	Agar well diffusion	
*Salmonella typhimurium* ATCC 1311	Phloretin	*Malus domestica* (Suckow) Borkh.	Microbroth dilution	[115]
	Iconisaflavan	*Glycyrrhiza aspera* Pall.	Microbroth dilution	[129]
	Iconisoflaven	*Glycyrrhiza aspera* Pall.	Microbroth dilution	
	(3S)-Licoricidin	*Glycyrrhiza aspera* Pall.	Microbroth dilution	
	Licorisoflavan A	*Glycyrrhiza aspera* Pall.	Microbroth dilution	
	Topazolin	*Glycyrrhiza aspera* Pall.	Microbroth dilution	
	Glycycoumarin	*Glycyrrhiza aspera* Pall.	Microbroth dilution	
*Vibrio cholerae* CO6	Chrysoeriol-7-*O*-*β*-d-xyloside	*Graptophyllum grandulosum* Turrill	Microbroth dilution	[128]
	Luteolin-7-*O*-*β*-d-apiofuranosyl-(1→2)-*β*-d-xylopyranoside	*Graptophyllum grandulosum* Turrill	Microbroth dilution	
	Chrysoeriol-7-*O*-*β*-d-apiofuranosyl-(1→2)-*β*-d-xylopyranoside	*Graptophyllum grandulosum* Turrill	Microbroth dilution	
	Chrysoeriol-7-*O*-α-l-rhamnopyranosyl-(1→6)-*β*-d-glucopyranoside	*Graptophyllum grandulosum* Turrill	Microbroth dilution	
	Isorhamnetin-3-*O*-α-l-rhamnopyranosyl-(1→6)-*β*-d-glucopyranoside	*Graptophyllum grandulosum* Turrill	Microbroth dilution	
*Vibrio cholerae* NB2	Chrysoeriol-7-*O*-*β*-d-xyloside	*Graptophyllum grandulosum* Turrill	Microbroth dilution	
	Luteolin-7-*O*-*β*-d-apiofuranosyl-(1→2)-*β*-d-xylopyranoside	*Graptophyllum grandulosum* Turrill	Microbroth dilution	
	Chrysoeriol-7-*O*-*β*-d-apiofuranosyl-(1→2)-*β*-d-xylopyranoside	*Graptophyllum grandulosum* Turrill	Microbroth dilution	
	Chrysoeriol-7-*O*-α-l-rhamnopyranosyl-(1→6)-*β*-d-glucopyranoside	*Graptophyllum grandulosum* Turrill	Microbroth dilution	
	Isorhamnetin-3-*O*-α-l-rhamnopyranosyl-(1→6)-*β*-d-glucopyranoside	*Graptophyllum grandulosum* Turrill	Microbroth dilution	
*Vibrio cholerae* PC2	Luteolin-7-*O*-*β*-d-apiofuranosyl-(1→2)-*β*-d-xylopyranoside	*Graptophyllum grandulosum* Turrill	Microbroth dilution	
	Chrysoeriol-7-*O*-*β*-d-apiofuranosyl-(1→2)-*β*-d-xylopyranoside	*Graptophyllum grandulosum* Turrill	Microbroth dilution	
	Chrysoeriol-7-*O*-α-l-rhamnopyranosyl-(1→6)-*β*-d-glucopyranoside	*Graptophyllum grandulosum* Turrill	Microbroth dilution	
	Isorhamnetin-3-*O*-α-l-rhamnopyranosyl-(1→6)-*β*-d-glucopyranoside	*Graptophyllum grandulosum* Turrill	Microbroth dilution	
	Chrysoeriol-7-*O*-*β*-d-xyloside	*Graptophyllum grandulosum* Turrill	Microbroth dilution	
*Vibrio cholerae* SG24	Chrysoeriol-7-*O*-*β*-d-xyloside	*Graptophyllum grandulosum* Turrill	Microbroth dilution	
	Luteolin-7-*O*-*β*-d-apiofuranosyl-(1→2)-*β*-d-xylopyranoside	*Graptophyllum grandulosum* Turrill	Microbroth dilution	
	Chrysoeriol-7-*O*-*β*-d-apiofuranosyl-(1→2)-*β*-d-xylopyranoside	*Graptophyllum grandulosum* Turrill	Microbroth dilution	
	Chrysoeriol-7-*O*-α-l-rhamnopyranosyl-(1→6)-*β*-d-glucopyranoside	*Graptophyllum grandulosum* Turrill	Microbroth dilution	
	Isorhamnetin-3-*O*-α-l-rhamnopyranosyl-(1→6)-*β*-d-glucopyranoside	*Graptophyllum grandulosum* Turrill	Microbroth dilution	

Viruses are microorganisms that cause both common and serious diseases in humans, including the common cold, influenza, chickenpox, rabies, and HIV. The threat of viruses to human health is still quite severe. Viral pandemics occurred over the past century, continuing into the present, and caused a substantial number of deaths and a lot of suffering. Examples include the influenza virus, the Ebola virus, the zika virus, and the SARS-CoV-2 virus. Vaccines are utilized for the prevention of viral infection, while antiviral drugs are used to treat the infected people. Globally, scientists are seeking effective antiviral drugs made from synthetic, semi-synthetic, and natural compounds. Studying flavonoids and polyphenols is attractive because of their antiviral activities. So, several scientific studies have investigated the antiviral activities of these phytochemicals. Firstly, the influenza virus, a highly infective agent, causes respiratory diseases, i.e., the common cold, flu, and acute pulmonary diseases, as well as seasonal epidemics worldwide [131]. Ling et al. studied the inhibition of epigallocatechin gallate extracted from green tea against the influenza virus in in vivo and in vitro experiments. They found that epigallocatechin gallate could suppress the replication of influenza A virus in a dose-dependent manner and improve the survival rate of mice infected with the influenza virus [132]. Lee et al. revealed that gallic acid derived from black raspberry (*Rubus coreanus* Miq.) exhibited antiviral activities against both influenza type A and B virus through the disruption of viral particles [133]. 

Similarly, ellagic acid is effective in combatting against influenza virus, including an oseltamivir-resistant strain [134]. In addition, procyanidin B2-di-gallate, a main active component of the aerial part of *Rumex acetosa* L., can inhibit the attachment and penetration of influenza A viruses [135]. Secondly, the herpes simplex virus is one of viral infectious diseases considered to be a health problem worldwide. There are 2 types of herpes simplex virus, namely, type 1, linked to oral infection, and type 2, associated with genital infection. De Oliveira and coworkers investigated the anti-herpes simplex type 1 effects of theaflavin polyphenol in black tea. They found that theaflavin digallate, at a concentration of 50 µm, was effective in terms of inhibiting the production of herpes simplex-1 viral particles [136]. According to the comprehensive review of Annunziata et al., resveratrol is a remarkable phytochemical displaying antiviral activity against the herpes simplex virus [137]. Furthermore, other flavonoids and polyphenols showed anti-herpes simplex virus activity were reported, such as 7-galloyl catechin, gallic acid, kaempferol, kaempferol 3-*O*-*β*-(6″-*O*-galloyl)-glucopyranoside, quercetin, and quercetin 3-*O*-*β*-(6″-*O*-galloyl)-glucopyranoside [138]. Thirdly, hepatitis C virus, the virus causing chronic cirrhosis and hepatocellular carcinoma, is a serious health problem, with approximately 130 million people chronically infected worldwide [139]. 

Interestingly, flavonoids and other polyphenols derived from tea (*C. sinensis* L.) demonstrated anti-hepatitis C virus potential. Examples include epigallocatechin gallate [139,140], 7,8-benzoflavone [140], and theaflavins and their derivatives (theaflavin-3′-monogallate and theaflavin-3-3′-digallate) [141]. Last but not least, severe acute respiratory syndrome coronavirus 2 (SARS-CoV-2) is a strain of coronavirus causing COVID-19 (coronavirus disease 2019). Jang and colleagues revealed that EGCG showed inhibitory activity towards SAR-CoV-2 3CL-protease, with an enzyme cleaving the viral polyprotein responsible for protein functions [142]. Furthermore, EGCG could reduce the quantity of coronavirus RNA and proteins in infected cell media [143]. The polyphenolic compounds—namely, broussochalcone A, papyriflavonol A, 3′-(3-methylbut-2-enyl)-3′,4′,7-trihydroxyflavane, broussoflavan A, kazinol F, and kazinol J—extracted from *Broussonetia papyrifera* (L.) L’Hér. ex Vent. interacted well with the catalytic residues (His41 and Cys145) of Mpro and displayed binding affinity (7.6 to 8.2 kcal/mol) [144]. According to the study of Agrawal et al., quercetin was able to intervene in the coronavirus entry and replication cycle, and so quercetin was suggested as a lead compound to be examined in future studies [145]. 

Approximately one billion people currently suffer from severe fungal diseases [146], which are thought to cause over 1.7 million annual fatalities. This was especially true in 2020 [147]. Serious fungal infections result from various medical conditions. such as AIDS, cancer, organ transplantation, and immunosuppressive and corticosteroid therapies [148]. Because of the rise in the number of fungal-infected patients and fungal antibiotic resistance, the novel and strong antifungal medications are urgently needed [149]. Since *Candida* species are the most prevalent fungi causing fungus infections in humans, there is a lot of scientific evidence showing antifungal activity against these fungal pathogens [150]. Quercetin is an eminent flavonoid exhibiting antifungal activity against *C. albicans*, a fungal pathogen linked to a number of clinical disorders and fatal systemic illnesses in humans [151]. Singh et al. reported that quercetin could combat fluconazole-resistant *C. albicans* NBC099 through the induction of apoptotic cell death [152,153]. In clinical therapy for vulvovaginal candidiasis brought on by *C. albicans* biofilm, quercetin proved to be an effective antifungal drug and a prospective synergist with fluconazole. [153]. 

In the case of *C. parapsilosis* (one of the main factors contributing to invasive candidal disease), quercetin also exerts fungicidal activity via a reduction in biofilm, and so it is suggested as a possible alternative means of controlling fungal biofilms [154]. Soberón et al. investigated the antifungal activity of *Tessaria dodoneifolia* (Hook. and Arn.) Cabrera, a medicinal plant traditionally used to treat fungal infection in northwestern Argentina. Naringenin and pinocembrin are two flavonoids, derived from *T. dodoneifolia* extracts, that show fungicidal activity against *C. albicans*; naringenin and pirocembrin function along with fluconazole to effectively combat *C. albicans*. Both are fluconazole-sensitive and -resistant [155]. Baicalein, a flavone derived from *Scutellaria baicalensis* Georgi root, demonstrated potent antifungal properties against *Aspergillus famigatus*, *Candida albicans*, *Trichophyton rubrum*, and *T. mentagrophytes* [156]. In several studies of flavonoids and polyphenolic compounds in relation to antifungal activity, flavonoids are frequently seen to inhibit the growth of fungi by a variety of mechanisms, including the breakdown of plasma membranes, the induction of mitochondrial dysfunction, and the inhibition of cell division, cell wall synthesis, RNA and protein synthesis, and the efflux-mediated pumping system [157]. So, flavonoids and other polyphenolics possess potential antifungal activity and, combined with conventional medication for the reinforcement of fungicidal effects and reductions in side effects and toxicity, are promising phytochemicals for application in therapeutic approaches. 

### 2.6. Cardioprotective Effect

Cardiovascular diseases (CVDs) are a cluster of disorders related to the cardiac system and blood vessels, such as coronary heart disease, cerebrovascular disease, deep vein thrombosis, peripheral arterial disease, rheumatic heart disease, and other conditions [158]. An estimated 17.9 million people die from CVDs, which are the main cause of death worldwide according to a WHO report in 2022 (https://www.who.int/health-topics/cardiovascular-diseases#tab=tab_1, accessed on 22 October 2024). Currently, modern medicines, i.e., antihypertensive, antidiabetic, and lipid-lowering drugs, are the main therapeutic means of preventing CVDs; however, patients suffer from their adverse effects [158]. Therefore, natural compounds have been alternatively chosen due to their cardioprotective effects. Flavonoids exhibit their positive outcomes through a number of mechanisms, including antioxidant and hypocholesterolemic properties, as well as modulatory impacts on numerous cells signaling pathways and gene expression [159]. In the case of quercetin, Ferenczyoma et al. studied the potential of quercetin in cardioprotection, and found a lot of scientific evidence in in vitro and ex vitro research as well as in vivo and ex vivo studies of cardiac injury, which demonstrated the cardioprotective effects of quercetin [160]. 

Furthermore, Bartekova et al. found that quercetin displayed an improvement in the post-ischemic recovery of left ventricular developed pressure as well as the conversion of contraction and relaxation markers [161]. Thereby, quercetin also showed beneficial effects to the people suffered from ischemia–reperfusion injuries. In the case of naringin, a citrus flavanone, cardioprotective effects have been discovered by various researchers. To investigate their cardioprotective potential, doxorubicin-induced cardiotoxicity was used as an important model for cardioprotection in animal studies. Naringin showed positive effects, achieving a decline in toxicity in this model [162,163]. Liu et al. demonstrated that the combination of doxorubicin and naringin could more effectively inhibit the cell proliferation than the use of these drug by themselves, and could also reduce the toxicity of doxorubicin [164]. According to epidemiological research, a diet high in flavonoids from fruits and vegetables is considered to promote health and delay or prevent the beginning of coronary heart disease [165]. The mechanisms by which cocoa flavonoids exerted their cardioprotective effects include a reduction in oxidative stress, the inhibition of platelet aggregation and low-density lipoprotein oxidation, the vasodilation of blood vessels, the inhibition of monocyte adhesion to the vascular endothelium, the promotion of fibrinolysis, and immunomodulatory and anti-inflammatory activity [166]. 

### 2.7. Neuroprotective Effect 

In both in vitro and in vivo settings, the natural polyphenol compounds found in various traditional medicines and medicinal plant (Table 2) sources possess antioxidant and anti-inflammatory properties, which aid in the achievement of neuroprotective effects through several mechanisms [167,168,169,170,171,172], such as the stimulation of endogenous antioxidant molecule synthesis in cells via the activation of the Nrf/ARE pathway [173], the modulation of a number of signaling pathways, namely NF-ĸB and SIRT [168], and the exertion of pleiotropic activity on cells [174]. There are various scientific studies demonstrating the neuroprotective effect of polyphenolic substances retrieved from botanical sources. Resveratrol, a stilbene-based compound found in the peel and seed of grapes, in peanuts, in blueberries, in bilberries, and in cranberries [175], produced a neuroprotective effect in in vitro and in vivo settings, including in clinical studies [176]. Several studies have shown the dominant neuroprotective effects of resveratrol. In clinical studies of the neuroprotective effect of *trans*-resveratrol on mild to moderate Alzheimer disease cases, the results demonstrated that it is beneficial for treating the disease. This is probably related to a reduction in neuroinflammation, including the accumulation and toxicity of amyloid-β in the brain [177]. Secoisolariciresinol is part of a group of lignans occurring in plants, particularly flax seed [178]. Rom et al. investigated the effects of secoisolariciresinol on the brain endothelium using cellular and animal models [179]. They reported that secoisolariciresinol exhibited anti-inflammatory and blood–brain barrier-protective effects in neuroinflammation cases. In 2020, there was a report on the potential of secoisolariciresinol to reduce dopamine neurodegeneration, induced by 6-hydroxydopamine, and lower amyloid-β toxicity in a *Caenorhabditis elegans* model [180]. In the flavonoid class, there are several scientific studies using cellular and animal models to demonstrate that flavonoids, namely, flavones, flavonols, flavanols, flavanones, isoflavones, and anthocyanins, possess neuroprotective effects [12,13,181]. Many studies showed the role of the flavonoids in ameliorating the symptoms in neurodegenerative disorders via the modulatory activities of neurotoxic agents such as oxidative stress [182,183,184] and related inflammatory cascades [184,185,186,187], including amyloid-β peptides [183]. In case of flavonols, quercetin is a key flavonol and it is extensively found in fruits and vegetables. Several scientific studies have demonstrated that quercetin possesses neuroprotective effects in both in vitro and in vivo models, counteracting cell toxicities and suppressing neuroinflammatory processes [188,189]. 

Interestingly, according to clinical trials and epidemiological studies, the consumption of plants containing high amounts of polyphenol or flavonoids can reduce the risk or incidence of neurodegenerative diseases. For example, according to a cross-sectional study conducted in Japan and China, both studies reported that the consumption of green tea containing various polyphenolic substances produced strong antioxidant effects, and resulted in a lower prevalence of cognitive impairment in Japanese older persons [190,191]. In case of grape consumption, Lee et al. performed a double-blind placebo-controlled pilot study to determine the impact of grape consumption on brain metabolism; the results pointed out the improvement in cognitive function in patients who suffered from mild declination of cognition [192]. 

**Table 2:** Neuroprotective potential of plant-derived flavonoids and polyphenols.

Flavonoids and Polyphenols	Sources	Neuroprotective Activities	References
Agathisflavone	Brazil’s Caatinga bean (*Poincianella pyramidalis*)	- stimulating neuron generation and improving the neuroprotective activity of microglia and astrocytes through estrogen signaling	[193]
Cannflavin A	*Cannabis*	- protecting amyloid β-mediated neurotoxicity related to the inhibitory activity of amyloid β fibrillisation	[194]
EGCG	green tea	- protecting the rat primary neurons from oxidative stress through inducing heme oxygenase	[195]
		- inhibiting *DYRK1A* gene which relate to brain morphogenesis	[196]
Fisetin	apple, strawberry	- increasing both pre- and postsynaptic protein levels to improve amyloid β1–42-induced synaptic dysfunction - preventing neuroinflammation through the suppression of many neuroinflammatory mediators	[197]
Hesperidin	sweet orange, lemon	- reducing lethality in NMDA-treated mice, decreased the behavioral signs of NMDA neurotoxicity, prevent oxidative stress in the brain of mice	[198]
Honokiol and magnolol	Magnolia	- decreasing amyloid-β-induced death of PC12 cells through reduced ROS production, suppression of intracellular calcium elevation, and inhibition of caspase-3 activity	[199]
		- preventing age-related learning and memory impairment by serving cholinergic neurons in the forebrain of senescence-accelerated mice (SAMP8)	[200]
		- inhibiting microglia NO production, microglia expression of pro-inflammatory cytokines, and multiple signaling pathways	[201]
Obovatol		- augmenting the level of paraoxonase 2 (PON2), a mitochondria enzyme expressed in brain cells, though the activation of JNK/AP-1 pathway	[202]
Quercetin	onion, capers	- decreasing reactive oxygen species (ROS) production and prevent H_2_O_2_-induced nuclear condensation - raising the activity of caspase 3/7 and poly (APD-ribose) polymerase expression	[203]
		- improving lipopolysaccharide (LPS)-induced neurotoxicity such as neuroinflammation-mediated neurodegeneration and synaptic/memory dysfunction in adult mice	[189]

### 2.8. UV-Protective Effect

The skin can be harmed by excessive ultraviolet (UV) radiation. The biological effects of excessive solar radiation on the skin include disorders such as the thickening of the stratum corneum, dermatitis, and sunburn, which are early responses to the destructive action of UVB (300–320 nm) [204]. In case of destructive UVA radiation (320–400 nm), it is mostly responsible for the delayed responses that result in nucleic acid and protein structural changes at the cellular and tissue levels [205]. UV radiation has the potential to cause several acute and chronic diseases, including sunburn, edema, neoplastic tumors, hyperplasia, immunosuppression, photoaging, and skin cancer [204,206]. Although both physical and chemical approaches have been utilized for photoprotection, flavonoids and other polyphenolics are among the most interesting choices, possessing antioxidant activity and exhibiting photoprotective effects. The mechanisms of their photoprotective properties are filtering solar radiation, reinforcing the endogenous antioxidant system, promoting DNA damage repair and melanogenesis, and inhibiting error-prone repair [207]. 

Catechins, mainly found in tea (*C. sinensis* and *C. assamica*), could promote photostability and protect the skin from UV radiation [208,209]. Wu et al. revealed that catechin was effective in preventing the death of UVB-induced human keratinocytes through the inhibitory activity of JNK phosphorylation [210]. Furthermore, silymarin and silybin, abundantly found in milk thistle (*Silybum marianum* (L.) Gaertn.), could reduce the amount of single-strand breaks and reactive oxygen species produced in response to UVA exposure, prevent glutathione depletion, and decrease the levels of matrix metalloprotective-1 protein and caspase-3 activation [211]. Apigenin from chamomile, thyme, onions, and spices displayed antioxidant capacity and photoprotective activity against UVA and UVB in studies of human keratinocytes [212]. According to these examples of photoprotective activity, flavonoids, other polyphenols, and flavonoid-rich plant extracts were chosen to develop oral photoprotective products and skin care products [213,214,215,216]. 

## 3. The Quality Evaluation of Traditional Medicines

Traditional medicinal recipes are the sources of flavonoids and other polyphenols aside from medicinal plants [81,82,95,99,102,134,135,181,217]. Humans have long used traditional medicines, using them since ancient times, especially in Asian countries [82,95,99,102,134,181,217]. Therefore, a quality evaluation needs to be carried out on the selected traditional medicinal recipes which will be future potential sources/raw material of flavonoids and other polyphenols. The quality of herbal drugs directly or indirectly affects the product’s safety, effectiveness, and acceptability [217,218,219,220,221,222,223,224,225,226,227,228]. The commercial herbal medicines are used in the form of fresh herbs, processed dried herbs, extracts, and herbal products from single or multiple species [221]. Furthermore, heavy metal and pesticide residues, as well as microorganism contaminations, probably contribute to several health problems that are intensively necessary to study [219]. Unlike modern medicine, herbal medicines still encounter several challenges, including herbal misidentification [220], substitution and adulteration [229], inconsistent composition [218], poor-quality products [230], and occasional cases of intoxication by adulterants and/or toxic components [231]. So, the national regulatory standards for herbal medicine, including quality control, were set up in various countries to manage quality assurance. The bodies include the American herbal pharmacopeia [232], the Chinese pharmacopeia [233], the Ayurvedic pharmacopeia of India [234], the Russian pharmacopeia [235], and the Thai herbal pharmacopeia [236]. Moreover, following the international collaborations between the World Health Organization (WHO) and scientists, the WHO launched the publication “Quality control methods for herbal materials” in order to assist in developing quality standards and requirements for herbal materials as part of the larger effort to ensure the efficacy and safety of herbal medicines [237]. 

International pharmacopeias and specifications provide documentation on analytical methods for quality control and the analysis of recognized herbal medicines. The monographs in pharmacopeia are different depending on the countries. However, individual monographs in these compendia provide the best practice standards of pharmacognostic analysis including the macro- and microscopical identification of plant species to assess physico-chemical parameters and for the chemical analysis of active ingredients [217,224,237,238,239]. Correct identification and the authentication of medicinal plants and traditional medicines are essential for achieving satisfactory efficacy, therapeutic properties, and the safety of the products [240]. Conventional methods, including macroscopic identification conducted using the characteristics of shape, size, color, texture, and cross-section [240], as well as the perception of taste, sight, smell, and touch [241], were the beginning steps in quality checking. These approaches are simple, rapid, and easy, but rely upon personal experience and knowledge [240]. Although the macroscopic diagnostic is simple and quickly identifies and authenticates herbal medicine, this examination may fail when applied to a preparation containing multi-ingredient powdered samples [242,243]. However, there are various problems in chemical analysis for the quality control of herbal medicines, for example, the deficiency of chemical markers, the influence of solvent and environment (temperature and light), and the instability of chemical structures (epimeric mixture and conformation) [218]. 

Accordingly, additional methods are necessary to ensure the reliability of results. Since the introduction of DNA barcoding in 2003, this approach has facilitated a considerable amount of research on herbal identification [244]. The use of DNA barcoding for herbal authentication has contributed to understanding unlabeled species that pose a serious risk to human health by having allergenic potential, known or suspected toxicity, side effects, and/or negative interactions with other herbs, supplements, or prescription medications [245]. DNA barcoding has been included in national pharmacopeias, British pharmacopeias [246], and Chinese pharmacopeias for the quality control and authentication of herbal materials [246]. However, DNA barcoding has practical limitations in terms of authenticating herbal preparations with a single ingredient, and only for unprocessed plant material [228,247]. As aforementioned, each identification method has advantages and limitations; therefore, a combination of diagnostic approaches has been applied to reinforce the quality assurance of the targeted medicinal plant and traditional medicine recipes that are selected as the sources of flavonoids and other polyphenols [248,249,250,251].

## 4. Interesting Research Directions and Perspectives for Future Studies

There are a large number of flavonoids and other polyphenols that are found in both traditional medicine recipes and medicinal plant species, especially in Asia. However, the authentication, validation, and standardization of these natural sources are necessary fundamental steps for phytopharmaceutical product development and other medical applications. The authentication of raw materials help industrial sectors to obtain the correct traditional medicine recipe and correct species and the right part of medicinal plant that provides the best type and highest number of flavonoids and other polyphenols. This will decrease the cost of phytopharmaceutical/medical product development, which is reflected in a reasonable price for those products on the market that people can easily afford. The validation and standardization of flavonoid/polyphenol-rich extract and/or the isolated phytochemicals are also initial steps to enriching the efficacy of the developed phytopharmaceutical/medical products.

According to the application of flavonoids and other polyphenols for phytopharmaceutical and medical aspects, choosing the suitable form, e.g., the standardized extract or pure phytochemical compound, is a key factor to be taken into account. For example, the standardized flavonoid/other polyphenol-rich extract may be an interesting choice of cosmetic/cosmeceutical product due to its synergistic effect, whereas a single/pure bioactive molecule of flavonoids or other polyphenols may be better choices for drug delivery in the future development of medicines. Likewise, a suitable model of studies such as in silico, in vitro, and in vivo animal models as well as clinical trials should be employed at each level of study to discover the answer to each hypothesis effectively and reliably.

Furthermore, the toxicity evaluation of the established phytopharmaceutical/medical products, particularly modern drugs and new formulations of herbal medicines, should be performed in cases of both acute and chronic toxicity using suitable models to ensure the safety of customers/patients. Additionally, those phytopharmaceutical/medical products also need clinical trials with reliable group sizes and clinical practice guidelines to confirm the safety and efficacy of those developed products before launching them in the market or applying them with patients.

Last but not least, the value of future research on these bioactive molecules from traditional medicines as well as medicinal plants with potential for phytopharmaceutical and medical applications is that it will benefit local people in a country. Governments can use the results from these studies to set up agriculture policy to encourage local people to grow and cultivate these plants or produce traditional medicine recipes with good quality control and best practice. This will help to increase local people’s income to improve their life and sustainability, and also assist the phytopharmaceutical and medical industries to reduce the cost of importing raw materials from foreign countries.

## 5. Conclusions

**Figure 3:**
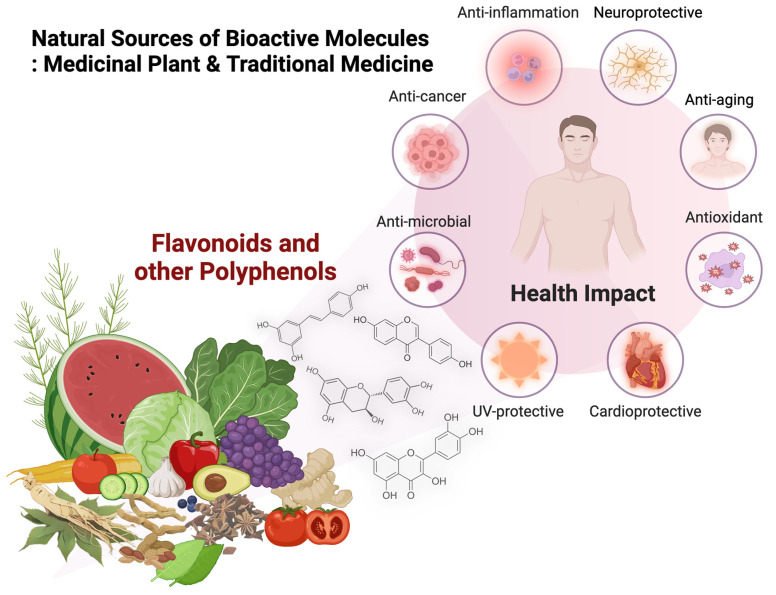
The summary on flavonoids and other polyphenols from traditional medicines/medicinal plants and their phytopharmaceutical and medical potentials. Created with BioRender.com/j25g033 by Aekkhaluck Intharuksa.

To recapitulate, flavonoids and other polyphenols from traditional medicines as well as medicinal plants have been of interest in the last decade to researchers seeking to discover their potential for medical and pharmaceutical benefits (Figure 3). Their potentials in terms of antioxidant, anti-aging, anti-cancer, anti-inflammatory, anti-microbial, cardioprotective, neuroprotective and UV-protective effects have been intensely studied in silico, in vitro, and in vivo using various animal models, but few clinical studies have evaluated their safety and efficacy with a trustworthy subject size and clinical practice guideline. Furthermore, modern drugs and the new formulation of herbal medicines from these bioactive ingredients should be investigated in terms of both acute and chronic toxicity to ensure their safety for customers/patients.
